# Efficacy of Chinese herbal medicine on poststroke depression in animal models: A systematic review and meta-analysis

**DOI:** 10.3389/fneur.2022.1095444

**Published:** 2023-01-09

**Authors:** Li Li, Bixiu Huo, Yuan Wang, Yao Wang, Ying Gong, Yun Zhang, Tingting Liu, Guiming Sha, Tianru Zheng

**Affiliations:** ^1^Elderly Demonstration Ward, Beijing Geriatric Hospital, Beijing, China; ^2^Hospital of Chengdu University of Traditional Chinese Medicine, Chengdu, China; ^3^Department of Pharmacy, Dongfang Hospital, Beijing University of Chinese Medicine, Beijing, China

**Keywords:** animal model, Chinese herbal medicine, depression-like behavior, meta-analysis, post-stroke depression

## Abstract

**Background:**

Poststroke depression (PSD) is a common complication that can seriously affect patients' functional recovery and quality of life after a stroke. Various side effects have been found to be associated with the pharmacological therapies used for PSD. Studies have shown that Chinese herbal medicine (CHM) can effectively improve PSD-like behavior and neurological function in clinical and animal studies. The efficacy of CHM on PSD in animal models has not been systematically analyzed.

**Methods:**

The following electronic databases were searched for articles published up to September 2022: PubMed, Web of Science, the Cochrane Library, and Embase. Studies that reported the efficacy of CHM in animals with PSD and were written in English were included. Depression-like behavior and the neurological deficit score were assessed as measures of efficacy. The included studies assessed depression-like behavior using sucrose preference, open-field, forced swimming, and tail suspension tests, as well as body weight. The Review Manager version 5.4 and STATA version 13.1 software packages were used for the meta-analysis. The standardized mean difference (SMD) with 95% confidence intervals was used to assess all the outcomes. Subgroup analyses were performed to explore the sources of heterogeneity. The Egger's test and funnel plots were used to assess the potential publication bias. Sensitivity analyses were used to identify the stability of the results.

**Results:**

A total of 14 studies, including 12 CHMs involving 442 rats, fulfilled the inclusion criteria for meta-analysis. The pooled results showed that CHM significantly alleviated neurological deficits (−1.72 SMD, −2.47– −0.97) and was efficacious in improving the depression-like behavior of rats in the sucrose preference (2.08 SMD, 1.33–2.84), open-field (2.85 SMD, 1.88–3.83), forced swimming (−1.83 SMD, −2.23−1.44), and tail suspension tests (−1.35 SMD, −1.94−0.76).

**Conclusion:**

Our results suggest that CHM could significantly improve depression-like behavior and neurological function in animals with PSD. The current results should be interpreted with caution because only animal studies were included.

## Introduction

Stroke remains the second-leading cause of death and the third-leading cause of death and disability combined (as expressed by disability-adjusted life-years lost) in the world (1). Poststroke depression (PSD) is the most common psychiatric disorder following a stroke (2). The prevalence of PSD is approximately 33% (3, 4). A review reported that the incidence of PSD within 2 years after stroke ranges from 11% to 41%. PSD is similar to major depressive disorder (MDD) but some symptoms differ (5, 6). Patients with PSD generally present with mood fluctuations, irritability, or apathy, while anhedonia, pessimism, suicidal ideation, or attention deficits are more common in patients with MDD. PSD negatively impacts rehabilitation following stroke, significantly increasing the chances of relapsing neurovascular events (3). PSD severely restricts the ability of patients to care for themselves and increases their dependence on other people in activities of daily living, which leads to poor quality of life (7). Moreover, PSD is associated with a significantly increased mortality risk in stroke survivors. A systematic review that included 15 prospective cohort studies, 250,294 participants, and 139,276 cases assessed the association between PSD and the risk of death and concluded that PSD increases all-cause mortality by 59% (8). Furthermore, the healthcare cost of patients with PSD is about four times that of poststroke patients without depression (9). Therefore, it is clear that PSD has serious economic impacts.

The pathophysiology of PSD is multifactorial and unclear. It probably involves dysregulation of the hypothalamic-pituitary-adrenal axis, increased inflammatory factors, decreased levels of monoamines, glutamate-mediated excitotoxicity, and abnormal neurotrophic response (10). The treatments for PSD include medication, psychotherapy, social intervention, and repetitive transcranial magnetic stimulation (11). Tricyclic antidepressants, selective serotonin reuptake inhibitors (SSRIs), serotonin and noradrenaline reuptake inhibitors, and monoamine oxidase inhibitors are the most common antidepressant drugs used (10). SSRIs are the first-choice drugs used to treat PSD, and tricyclic drugs should be considered for second-line therapy (4). However, almost all antidepressant drugs are associated with adverse effects. A large cohort study suggested that all commonly used antidepressants can contribute to significantly increased risks of adverse outcomes, including mortality, suicide, hemorrhagic complications, falls, and upper gastrointestinal bleeding (12–14). High-quality evidence suggests that SSRIs increased the risk of bone fractures and possibly the risk of seizures (15). Therefore, other treatment options need to be explored.

Traditional Chinese medicine, including Chinese herbal medicine (CHM), acupuncture, moxibustion, and Tuina, has long been used to manage various disorders in China (16). A series of animal studies (17–21) have shown that traditional Chinese medicine can improve depression-like behavior and neurological function of PSD rats by regulating the signaling pathways in the neurotrophic pathway and reducing neuroinflammatory responses. However, the efficacy of CHM in PSD animals has not been systematically reviewed. Here, we performed a systematic review and meta-analysis to comprehensively review the efficacy of CHM in treating PSD based on animal studies.

## Methods

We performed this systematic review and meta-analysis in accordance with the Preferred Reporting Items for Systematic Reviews and Meta-Analyses guidelines (22). The efficacy outcomes assessed include the neurological deficit score, body weight, as well as sucrose preference, open-field, forced swimming, and tail suspension test results. Low body weight, less sugar consumption in the sucrose preference test, longer immobility in the forced swimming and tail suspension tests, and less movement in the open-field test were considered to be signs of depression.

### Search strategy

Studies published up to 25 September 2022 were retrieved from the Web of Science, PubMed, Cochrane Library, and Embase databases. Studies that reported the efficacy of CHM in animals with PSD were included. The literature search was conducted using the terms (“depression”) AND (“stroke”) AND (“animal” OR “animals” OR “rat” OR “rats” OR “mouse” OR “mice”) AND (“Chinese medicine” OR “Chinese herb” OR “herbal” OR “natural drug” OR “Natural Product” OR “traditional Chinese” OR “formula”). Only publications written in English were included in this review. All searches were performed by two independent researchers (TL and GS).

### Inclusion and exclusion criteria

The inclusion criteria were as follows: (1) studies that reported the efficacy of CHM in PSD animal models; (2) studies that used CHM, including Chinese herbal compounds, a single Chinese herbal extract (monomer), and compounds of several Chinese herbal extracts; (3) studies that mentioned the number of animals used; and (4) studies that were published in English.

The exclusion criteria were as follows: (1) studies that included an agent that was a natural plant extract but not the main ingredient in traditional CHM; (2) studies where the data could not be obtained. Studies were independently screened by two researchers (LL and YG), and any disagreements were resolved through discussion.

### Data extraction

Two investigators (LL and BH) independently extracted data from the included studies. We extracted data on the outcome measures (neurological deficit score and body weight as well as the sucrose preference, open-field, forced swimming, and tail suspension test results), publication details (author and year), treatments used (route and dose), and animals (species) from the studies. For each comparison, the sample size, mean value, and standard deviation (SD)/standard error for both the treatment and control groups were extracted. If multiple treatment groups shared the same control group, the sample size of the control group was divided by the number of treatment groups (23). If outcomes were measured at more than one time point, we only included data from the last time point (24). If outcomes were presented graphically, we used the ImageJ software (NIH, United States) to quantify the results.

### Risk of bias assessment

Two researchers independently assessed the quality of the included studies using the Systematic Review Center for Laboratory Animal Experimentation Risk of Bias tool. The assessment covered 10 areas, and each item was scored as one point as follows: (1) sequence generation, (2) baseline characteristics, (3) allocation concealment, (4) random housing, (5) blinding (for animal breeders and researchers), (6) random outcome assessment, (7) blinding (for the outcome evaluator), (8) incomplete outcome data, (9) selective outcome reporting, and (10) other sources of bias. Any disagreements arising from the evaluation were resolved through discussion until a consensus was reached.

### Subgroup analysis

There were no significant differences in the subjects, modeling, and administration methods used in the included studies. While the CHM used differed, it could be grouped into monomers and compounds. We performed a subgroup analysis on the different types (according to name) and forms (compound or monomer) of CHM to evaluate the influence of the variables or research characteristics on the estimated effect size.

### Data analysis

Depending on the heterogeneity test, data were synthesized using a fixed effect model or random effect model. Heterogeneity across studies was explored by Cochran's *Q* statistic and *I*^2^ statistic (25). The effect size was pooled using a random-effects model for the neurological deficit score, body weight, sucrose preference test results, and open-field test results. For the measured values of the forced swimming and tail suspension tests, the effect sizes were pooled using the fixed-effects model. We established a subgroup to explore the source of heterogeneity. The Egger's test and funnel plots (26) were used to assess the potential publication bias. Sensitivity analysis, performed by removing individual studies one at a time, was used to assess the stability of the results. All the data were analyzed using the Review Manager version 5.4 and STATA version 13.1 software packages.

## Results

### Study characteristics

**Figure 1:**
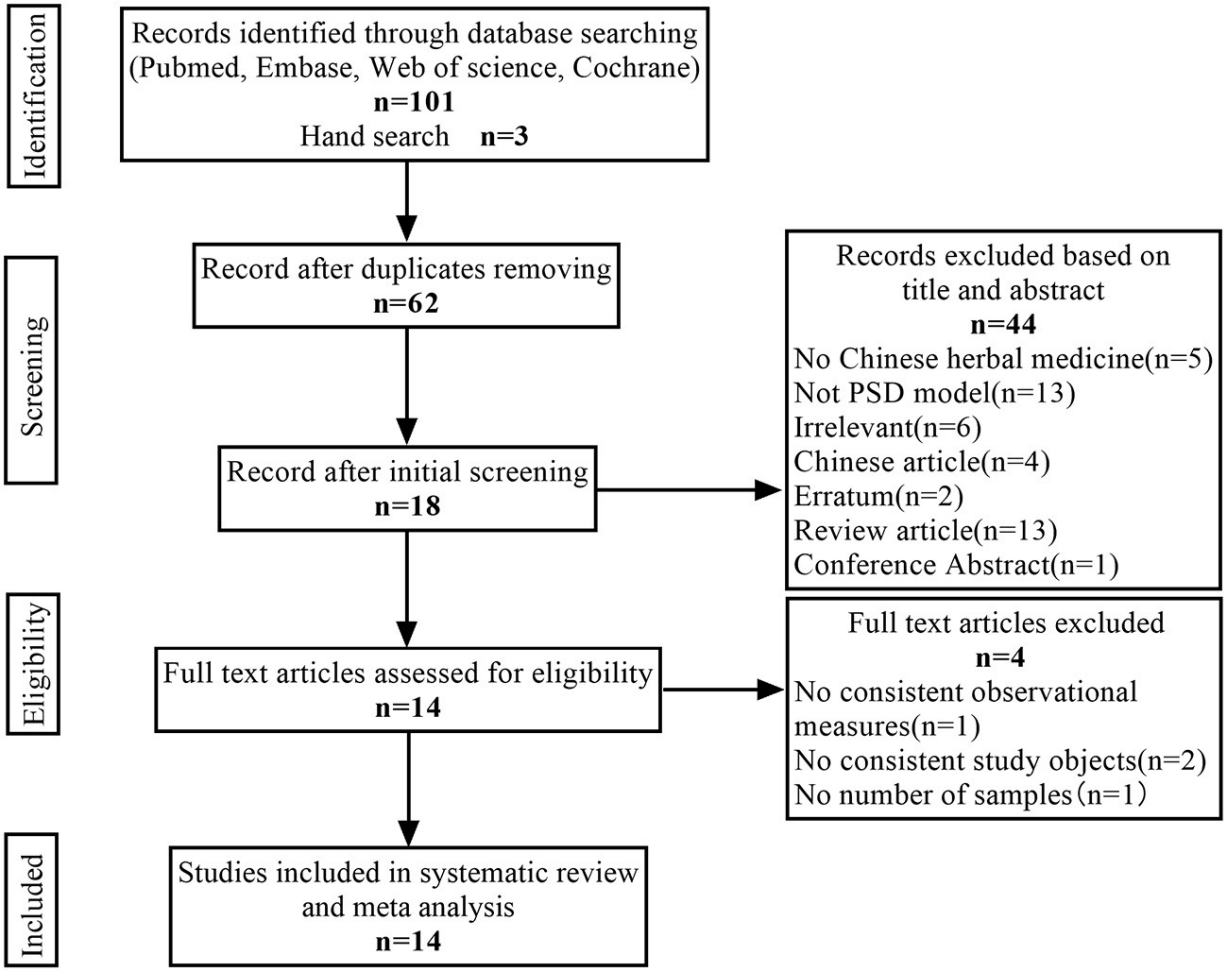
Flow diagram of the study selection process.

A total of 104 articles (20 from PubMed, 20 from Web of Science, 20 from Cochrane Library, 41 from Embase, and 3 from hand search) were found, 42 duplicate articles were excluded using Endnote X 9, and 44 articles were manually excluded according to the title and abstract. For the remaining 18 articles, four articles that did not meet the inclusion criteria were excluded after reading the full text. Therefore, 14 articles were finally included in our analysis. The specific screening procedure is shown in Figure 1.

Altogether, 442 animals (278 in the CHM group; 164 in the PSD group) were included in this meta-analysis. Twelve studies used Sprague Dawley rats, and two studies used Wistar rats. The weight of the rats varied between 155 and 320 g. Only four studies mentioned the age of the rats, which ranged from 7 to 24 weeks. There were five kinds of PSD models used in the included studies. Ten studies used middle cerebral artery occlusion combined with chronic unpredictable mild stress (CUMS). One study used unilateral carotid artery ligation combined with separate housing and a small dose of reserpine. One study used unilateral internal carotid artery ligation combined with solitary housing and CUMS. One study used carotid artery embolization combined with chronic sleep deprivation. One study used bilateral common carotid artery ligation combined with forced swimming.

Regarding the intervention methods, there were 12 types of CHM, including eight compounds and four monomers. The monomers included Morinda officinalis oligosaccharides (MOOs), peoniflorin, Saikosaponin A (SSA), and Huperzine A (HupA). The intervention durations ranged from 14 to 56 days. Intraperitoneal administration was performed in 2 of the 14 studies, and gastrointestinal administration was performed in the other 12 studies. Among the 14 included studies, 13, 12, 7, and 3 performed the sucrose preference, open-field, forced swimming test, and tail suspension tests, respectively, and seven and four measured body weight and neurological deficit scores, respectively. Among the 12 studies that conducted an open-field test, the effect sizes of three studies were inconsistent with the other nine studies. Therefore, we chose to analyze only nine of them. The detailed study characteristics are listed in Table 1.

**Table 1:** Characteristicsof included studies.

**References**	**CHM drug**	**Route**	**Duration (days)**	**Species**	**Weight (g)**	**Age (weeks)**	**Model**	***N*** ** (T/C)**	**Treat**	**Control**	**Possible mechanisms**	**Outcome measures**
Du et al. (27)	JYAS	i.g.	28	SD rats	240–260	7–9	MCAO+CUMS	10/10	1g/kg/d 3g/kg/d	NR	Modulation on monoamine system, neuroendocrine, neuroinflammation, and neurogenesis	SPT 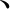 OFT
Fan et al. (28)	XNJY	i.g,	21	SD rats	NR	24	unilateral carotid artery ligation +separately housed +reserpine	10/10	45 mg/100g/d 15 mg/100g/d 7.5mg/100g/d	Saline	Increased the size and quantity of hippocampal neurons and glial cells with regular shape and decreased intercellular space	SPT 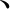 OFT
Li et al. (18)	XNJY	i.g,	21	SD rats	180 ± 20	NR	MCAO+CUMS	12/12	10.5g/kg/d 21g/kg/d	NR	Regulation of the BDNF/ERK/CREB signaling pathway.	BW 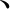 SPT OFT 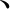 FST
Li et al. (19)	MOOs	Orally	14	SD rats	250–280	NR	tMCAO+CUMS	8/8	0.1 mg/g/d	Vehicle	Negatively regulate the microglial NLRP3 inflammasome activation	BW 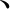 SPT OFT 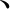 FST 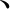 TST
Tian et al. (29)	YNJYP	Gavage	56	SD rats	300–320	7–9	MCAO+CUMS	6/6	9.92 g/kg/d	Saline	Dynamically regulating the expression of Notch signaling genes	SPT 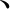 FST
Yan et al. (30)	XNJY	i.g,	28	SD rats	175 ± 20	NR	unilateral internal carotid artery ligation+solitary housing +CUMS	8/8	45mg/100g/d 15mg/100g/d 7.5mg/100g/d	NR	Upregulate synaptotagmin expression in hippocampi	SPT 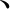 OFT
Wang et al. (21)	SSA	i.p.	24	SD rats	230–260	NR	MCAO + isolation + CUMS	10/10	5 mg/kg	Saline	Inhibited hippocampal neuronal apoptosis after cerebral ischemia through p-CREB/ BDNF pathway	BW 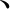 NDS 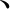 SPT 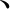 OFT 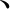 FST
Zhao et al. (31)	JDTLG	i.g.	28	SD rats	200–220	NR	carotid artery embolization+chronic sleep deprivation	10/10	2 g /kg/d 4 g /kg/d	Water	Reduce glutamate (Glu) level and increase gamma-aminobutyric acid (GABA) level via regulating the NMDAR/BDNF pathway	BW 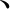 NDS 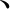 SPT 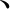 OFT 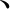 TST
Zhao et al. (32)	YNJYD	Gavage	28	Wistar rats	200 ± 10	6	MCAOS+CUMS	6/6	0.4 g/kg/d	Saline	Improving microcirculation in the PFC and HP, regulating glutamatergic systems and membrane phospholipid metabolism, and repairing microstructural damage	SPT 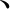 OFT
Zhu et al. (17)	MOOs	Orally	14	SD rats	250-280	NR	tMCAO+CUMS	8/8	0.1 mg/g/d	Vehicle	Upregulating GLUT3 to improve synaptic activity	BW 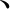 SPT 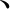 OFT 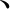 FST 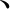 TST
Wang et al. (33)	BSYQ HXQY DTKQ	i.g.	56	Wistar rats	250 ± 50	NR	Deligating bilateral common carotid arteries permanently+forced swimming	8/8	BSYQ 18g/kg/d HXHY 9g/kg/d, DTKQ 9g/kg/d)	Saline	Regulating the expressions of c-Fos and c-Jun in hippocampus	OFT
Yan et al. (34)	XNJY	i.g.	21	SD rats	200 ± 20	NR	MCAOS+CUMS	12/12	10.5g/kg/d 21g/kg/d 42g/kg/d	Saline	Alleviating neuroinflammation	BW 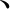 SPT 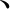 OFT 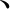 FST
Hu et al. (35)	PF	i.p.	21	SD rats	240–260	NR	MCAOS+CUMS	46/46	5mg/kg/d	Saline	Increased BDNF and p-CREB expression in the CA1 region	BW 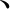 NDS 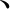 SPT 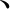 OFT
Du et al. (36)	HupA	i.g.	28	SD rats	240–260	NR	MCAO+CUMS	8/10	0.05 mg/kg/d 0.15 mg/kg/d	NR	Upregulate hippocampal expression of 5-HT1AR, p-CREB and BDNF, and increase levels of NE, DA, and 5-HT in the hippocampus and prefrontal cortex	NDS 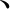 SPT 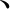 FST

JYAS, Jieyu Anshen; XNJY, Xingnao Jieyu; MOOs, Morinda officinalis oligosaccharides; YNJYP, Yi-nao-jie-yu Prescription; SSA, Saikosaponin A; JDTLG, Jiedu Tongluo Granules; YNJYD, Yi-nao-jie-yu Decoction; BSYQ, Bushen Yiqi; HXQY, Huoxue Huayu; DTKQ, Ditan Kaiqiao; PF, paeoniflorin; HupA, Huperzine A; i.g., intragastric injection; i.p., intraperitoneal injection; SD, Sprague Dawley; NR, not reported; MCAO, middle cerebral artery occlusions; tMCAO, transient middle cerebral artery occlusion; CUMS, chronic unpredictable mild stress; d, days; BW, body weight; NDS, neurological deficit score SPT, sucrose preference test; FST, forced swimming test; OFT, open field test; TST, tail suspension test; BDNF, brain-derived neurotrophic factor;EPK, extracellular signal-regulated kinase; CREB, cyclic adenosine monophosphate response element binding protein; NLRP-3, Nucleotide- binding domain leucine- rich repeat family pyrindomain containing 3; p-CREB, phosphorylated CREB; NMDAR, N-methyl-D-aspartate receptors; GLUT3, glucose transporter-3; 5-HT_1A_R, 5-hydroxytryptamine 1A receptor;NE, norepinephrine; DA, dopamine; 5-HT, 5-hydroxytryptamine.

### Risk of bias and quality of the included studies

Among the 14 included studies, no study described the method used to generate the allocation sequence. All studies reported similar baseline characteristics between the groups. No study clarified whether the allocation of different groups was sufficiently blinded. The breeding conditions and environments of all experimental animals included in the studies were the same. Therefore, we considered that the animal placements complied with the principle of randomization. No study reported sufficient information regarding the blinding methods used by the caregivers or investigators. A total of 11 studies assessed the randomization of the outcomes. Seven studies described the blinding methods used for the outcome evaluation. All studies reported complete outcome data and preliminary reported results and had no other sources of bias. A complete quality assessment of the included studies is shown in Table 2.

**Table 2:** Study quality score report.

**Author**	**Year**	**(1)**	**(2)**	**(3)**	**(4)**	**(5)**	**(6)**	**(7)**	**(8)**	**(9)**	**(10)**	**Aggregate quality score**
Du, Y	2020	–	+	–	+	–	–	–	+	+	+	**5**
Fan, WT	2012	–	+	–	+	–	+	–	+	+	+	**6**
Li, T	2018	–	+	–	+	–	–	+	+	+	+	**6**
Li, ZF	2021	–	+	–	+	–	+	–	+	+	+	**6**
Tian, HL	2018	–	+	–	+	–	–	–	+	+	+	**5**
Yan, YM	2013	–	+	–	+	–	+	–	+	+	+	**6**
Wang, AR	2021	–	+	–	+	–	+	+	+	+	+	**7**
Zhao, AM	2021	–	+	–	+	–	+	–	+	+	+	**6**
Zhao, ZJ	2020	–	+	–	+	–	+	+	+	+	+	**7**
Zhu, JY	2020	–	+	–	+	–	+	+	+	+	+	**7**
Wang, HY	2006	–	+	–	+	–	+	–	+	+	+	**6**
Yan, YM	2019	–	+	–	+	–	+	+	+	+	+	**7**
Hu, MZ	2019	–	+	–	+	–	+	+	+	+	+	**7**
Du, Y	2017	–	+	–	+	–	+	+	+	+	+	**7**

“+” means this part was reported in the study, “–” means not reported.

### Meta-analysis on the effects of CHM

#### Effects on depression-like behavior

**Figure 2:**
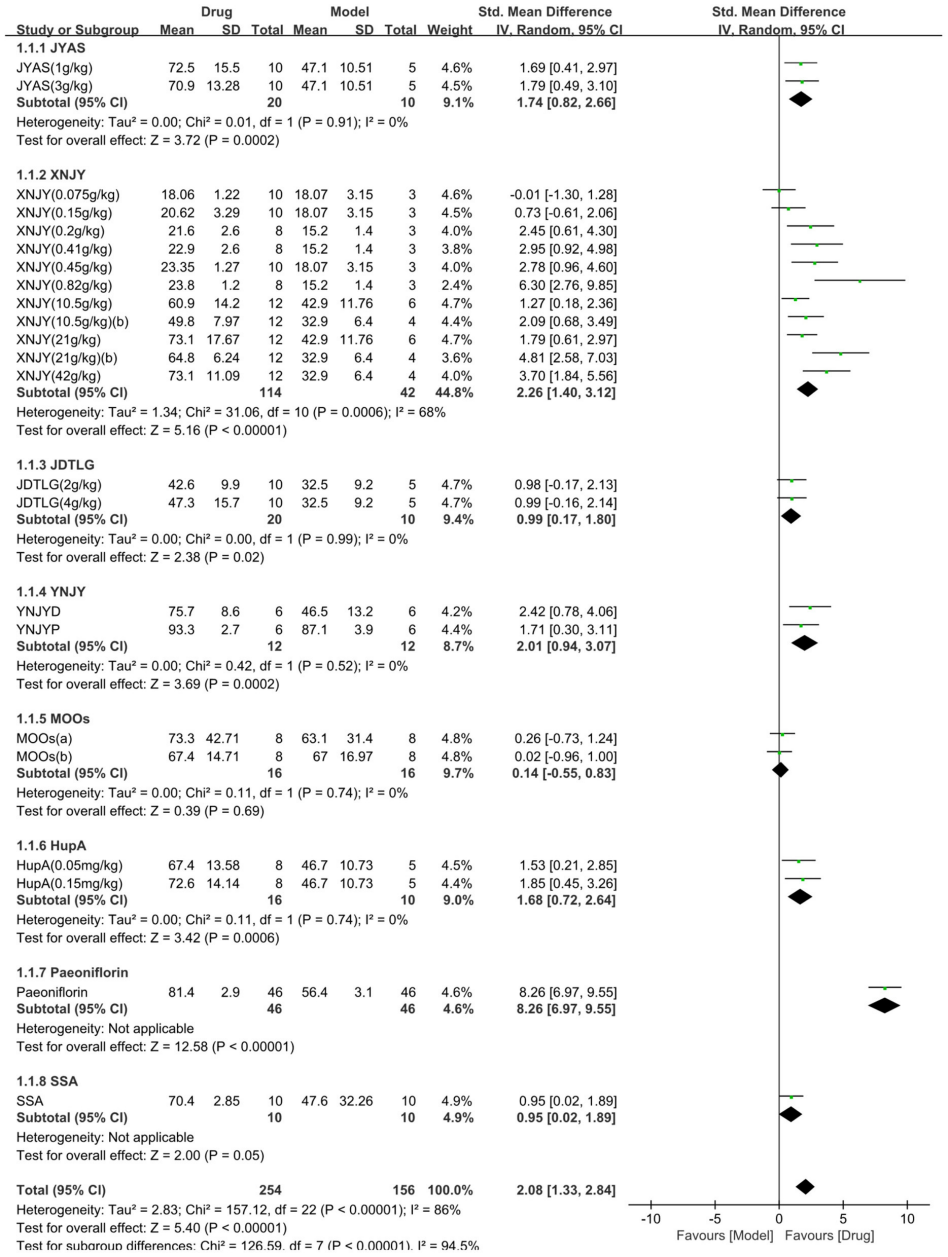
Efficacy of Chinese herbal medicine (CHM) on sucrose preference test. Forest plots of the effect size calculated using standardized mean differences (SMDs). The horizontal error bars represent the 95% confidence interval of individual studies.

The sucrose preference test was used in 13 studies (410 animals, 254 were in the CHM group and 156 were in the control group), which are included in the meta-analysis (Figure 2). The pooled results showed that CHM significantly increased sucrose preference by 2.08 SMD (95% CI, 1.33–2.84; *P* < 0.001; 23 comparisons), with substantial heterogeneity between studies (χ^2^= 157.12; *I*^2^= 86%; df = 22; *P* < 0.001). Subgroup analysis showed a significant correlation (*P* < 0.001). The effects of MOOs and SSA were similar to that of the control group (*P* = 0.69, *P* = 0.05, respectively). The effects of the other CHM on the sucrose preference test were more obvious than that of the control group (*P* < 0.05). The forest map showed that peoniflorin played a more prominent role.

**Figure 3:**
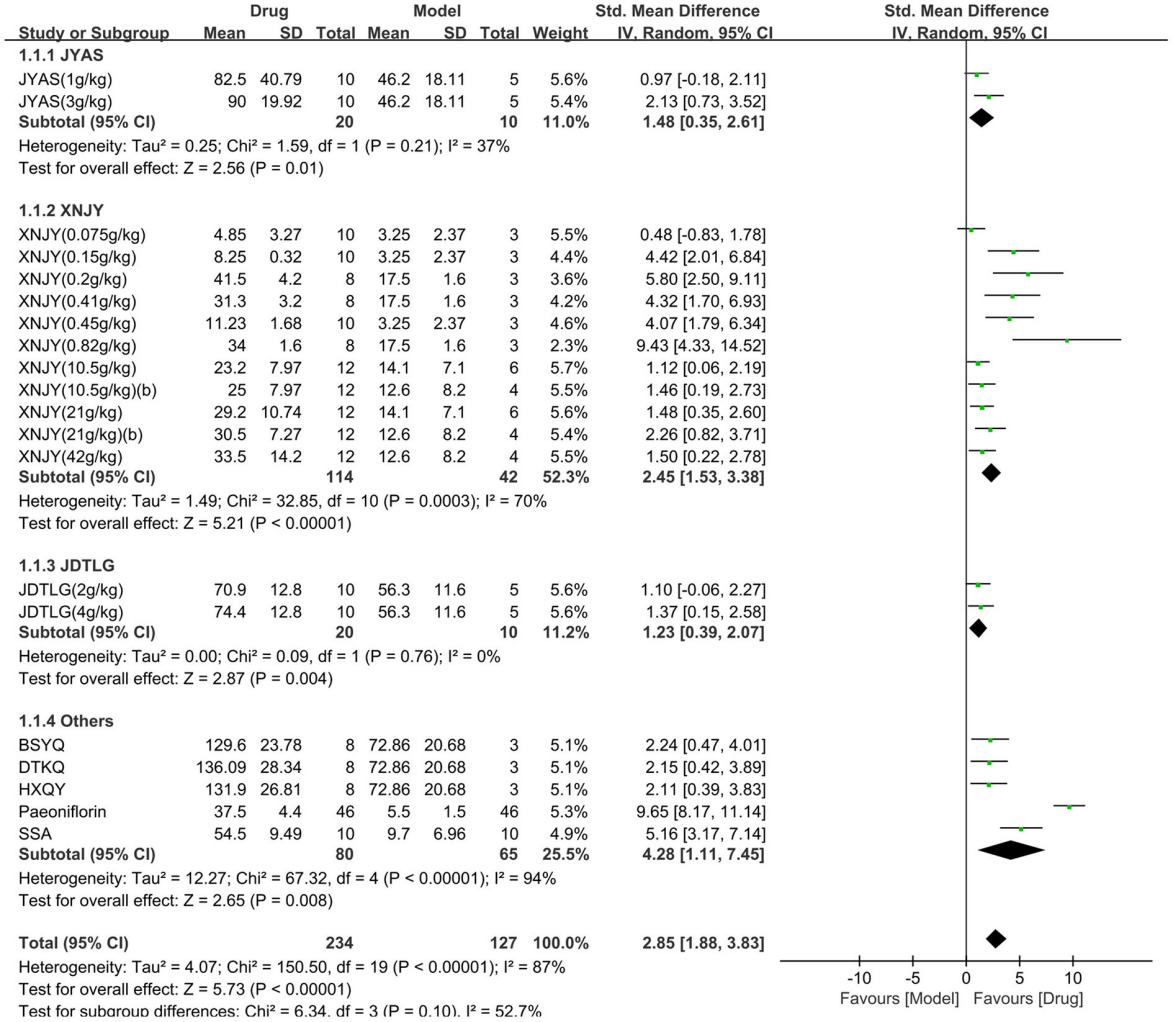
Efficacy of CHM on the open-field test. Forest plots of the effect size calculated using SMDs. The horizontal error bars represent the 95% confidence interval of individual studies.

The open-field test was used in nine studies (361 animals, 234 were in the CHM group and 127 were in the control group), which are included in the meta-analysis (Figure 3). The pooled results showed that the CHM group had significantly improved the movement in the open-field test compared to the control group by 2.85 SMD (95% CI, 1.88–3.83; *P* < 0.001; 20 comparisons), with substantial heterogeneity between studies (χ^2^= 150.5; *I*^2^= 87%; df = 19; *P* < 0.001). Subgroup analysis did not show any effect of the type of CHM on the results (*P* = 0.1), and each drug improved the movement in the open-field test (*P* < 0.05).

**Figure 4:**
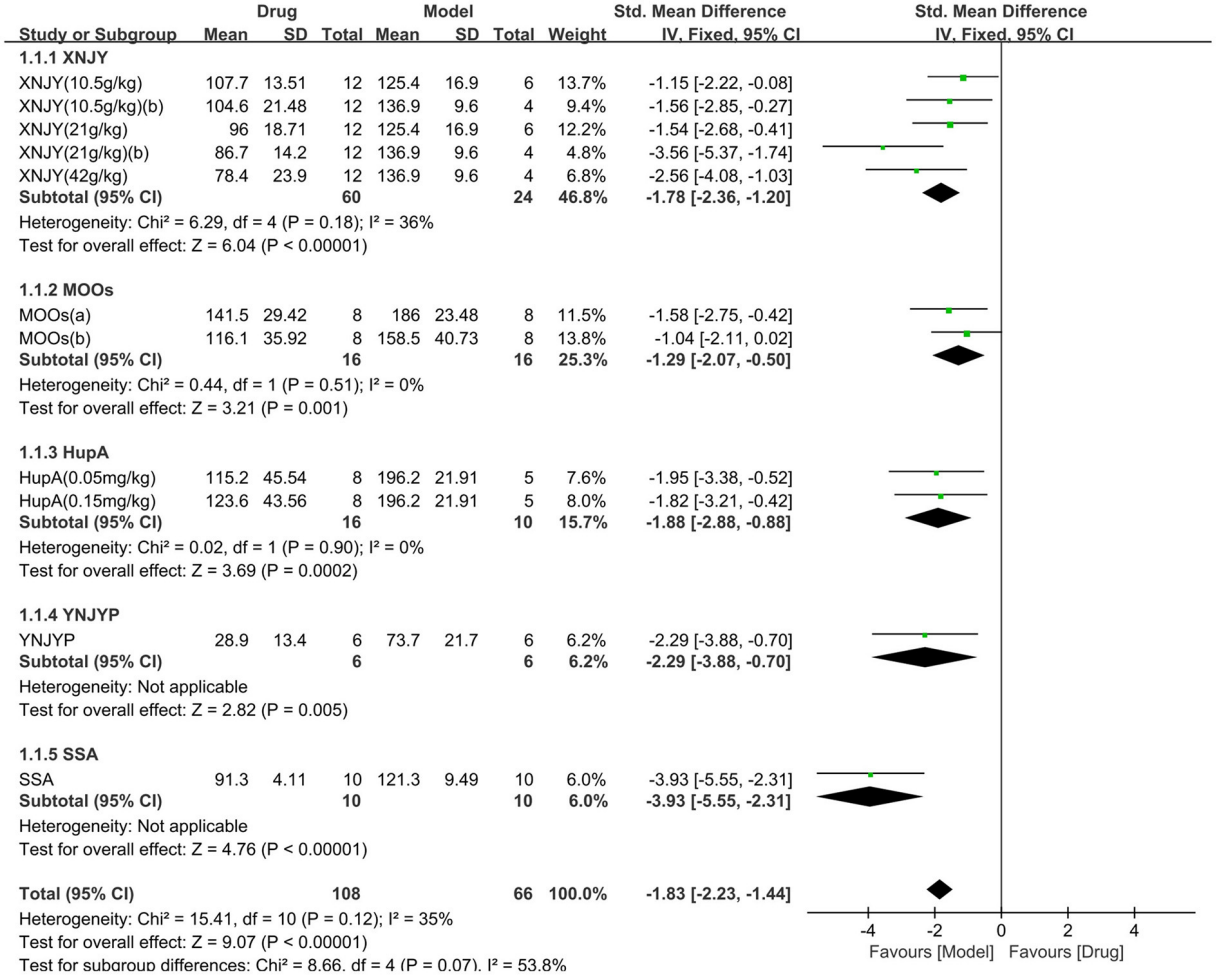
Efficacy of CHM on forced swimming test. Forest plots of the effect size calculated using SMDs. The horizontal error bars represent the 95% confidence interval of individual studies.

As for the forced swimming test, seven studies (174 animals, 108 were in the CHM group and 66 were in the control group) were included in the meta-analysis (Figure 4). The pooled results showed that CHM significantly decreased the immobility time of the forced swimming test by −1.83 SMD (95% CI, −2.23−1.44; *P* < 0.00001; 11 comparisons), with moderate heterogeneity between studies (χ^2^= 15.41; *I*^2^= 35%; df = 10; *P* = 0.12). No statistical significance was found when the CHM type was analyzed as a subgroup (*P* = 0.07), and each drug reduced the immobility time of the forced swimming test (*P* < 0.05). The forest map showed that SSA played a more prominent role.

**Figure 5:**
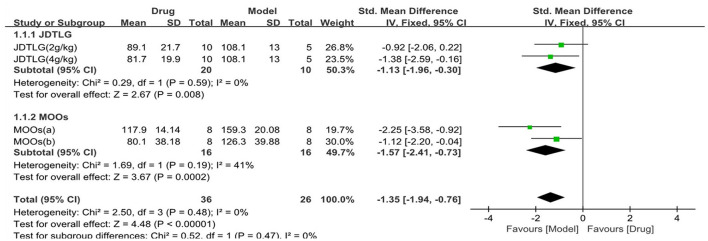
Efficacy of CHM on tail suspension test. Forest plots of the effect size calculated using SMDs. The horizontal error bars represent the 95% confidence interval of individual studies.

The tail suspension test was used in three studies (62 animals, 36 were in the CHM group and 26 were in the control group), which are included in the meta-analysis (Figure 5). The pooled results showed that the immobility time in the tail suspension test was significantly decreased in the CHM group compared to the control group by −1.35 SMD (95% CI, −1.94 to −0.76; *P* < 0.001; 4 comparisons), with no heterogeneity between studies (χ^2^= 2.5; *I*^2^= 0; df = 3; *P* = 0.48). Subgroup analysis did not show any effect of the type of CHM on the results (*P* = 0.47), and each drug reduced the immobility time in the tail suspension test (*P* < 0.05).

**Figure 6:**
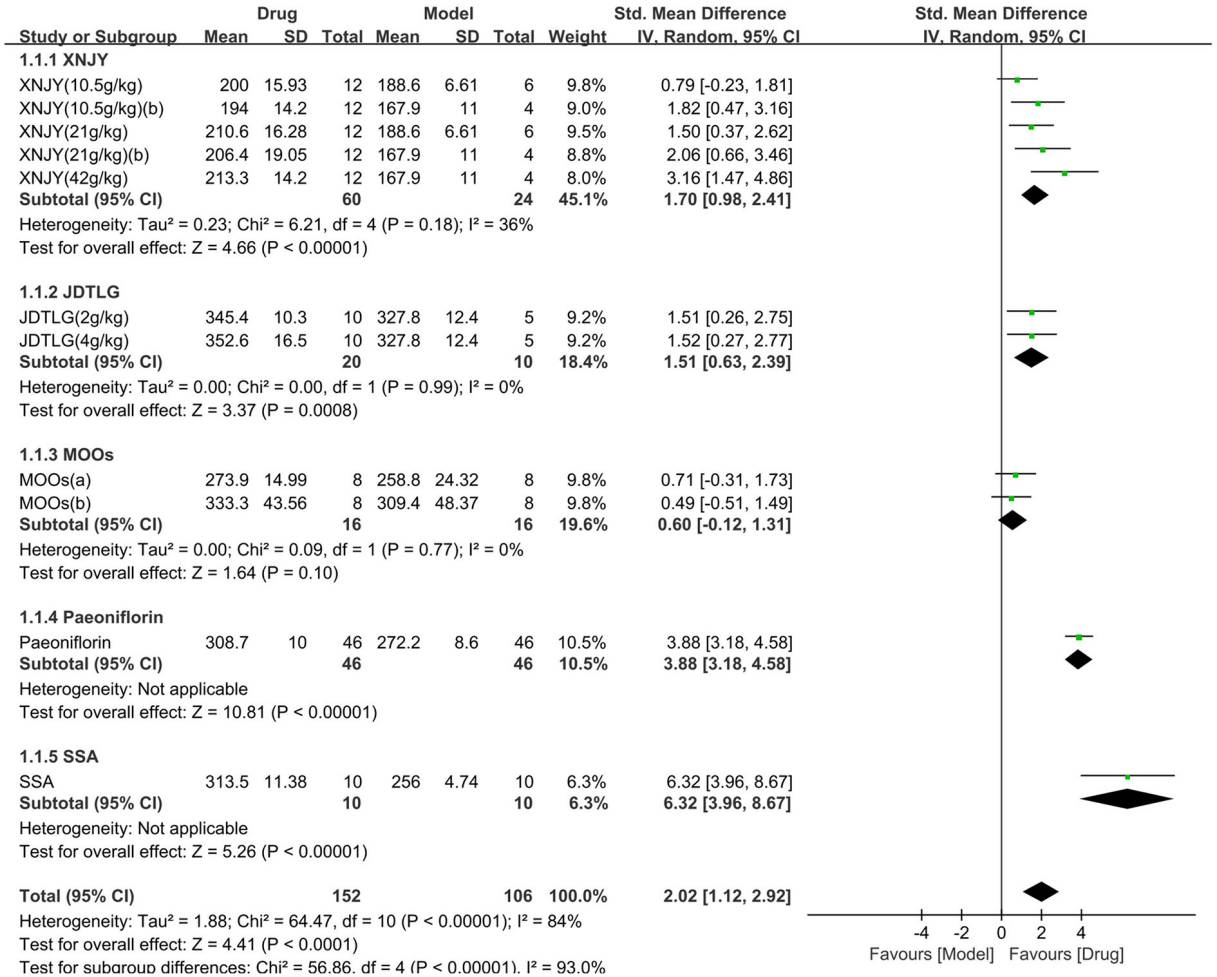
Efficacy of CHM on body weight. Forest plots of the effect size calculated using SMDs. The horizontal error bars represent the 95% confidence interval of individual studies.

Body weight was assessed in seven studies (258 animals, 152 were in the CHM group and 106 were in the control group), which are included in the meta-analysis (Figure 6). The pooled results showed that CHM significantly increased body weight by 2.02 SMD (95% CI, 1.12–2.92; *P* < 0.001; 11 comparisons), with substantial heterogeneity between studies (χ^2^= 64.47; *I*^2^= 84%; df = 10; *P* < 0.001). Subgroup analysis showed a significant correlation between the type of CHM and effect size (*P* < 0.001). The effect of MOOs on body weight was similar to the control group (*P* = 0.1), but the remaining CHM were all associated with significantly increased body weight (*P* < 0.05). The forest map showed that the effect of SSA and peoniflorin remained more obvious.

#### Effect on neurological deficit score

**Figure 7:**
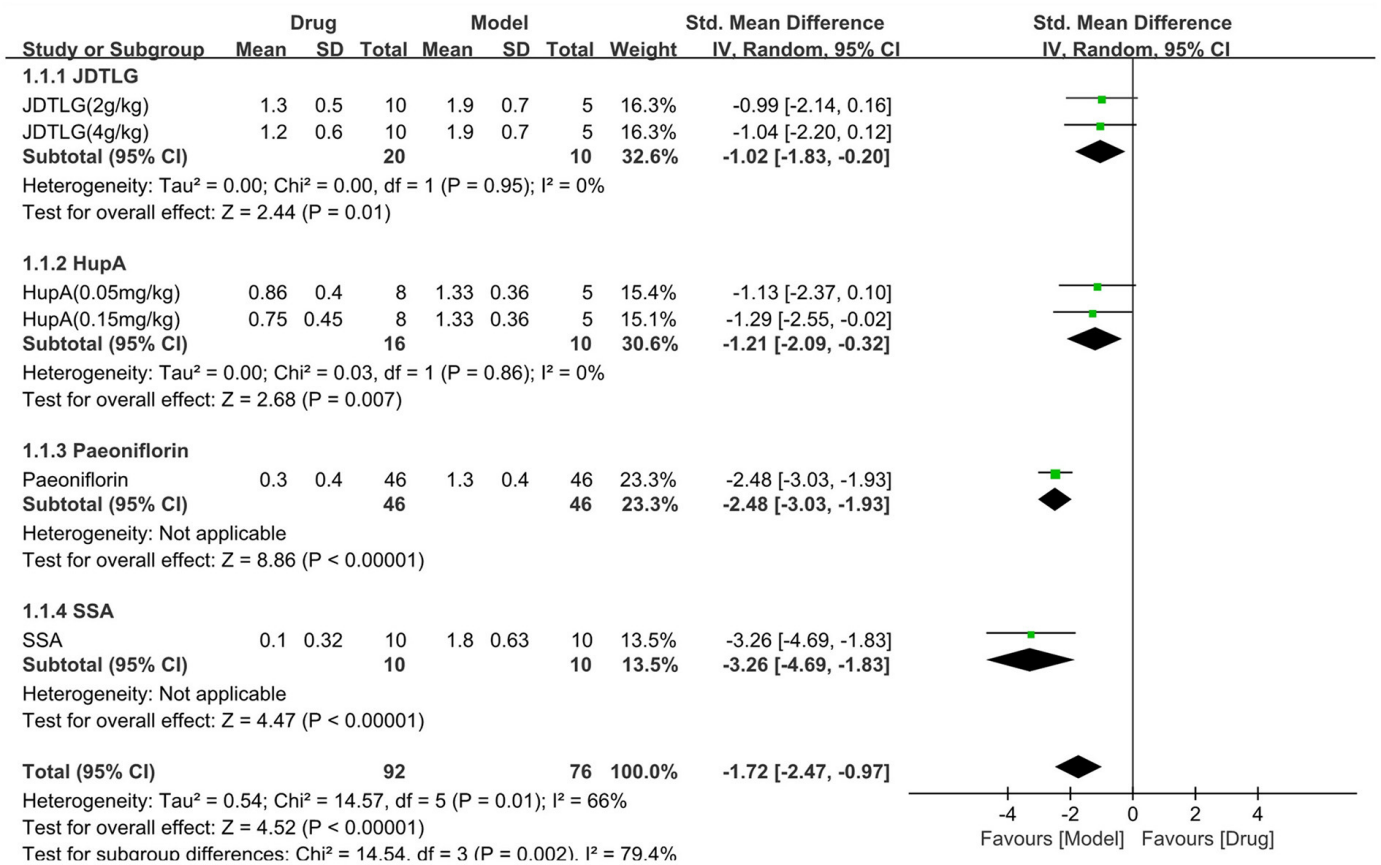
Efficacy of CHM on neurological deficit score. Forest plots of the effect size calculated using SMDs. The horizontal error bars represent the 95% confidence interval of individual studies.

The neurological deficit score was assessed in four studies (168 animals, 92 were in the CHM group and 76 were in the control group), which are included in the meta-analysis (Figure 7). The pooled results showed that the neurological deficit score of the CHM group was significantly lower than that of the control group by −1.72 SMD (95% CI, −2.47−0.97; *P* < 0.001; 6 comparisons), with substantial heterogeneity between studies (χ^2^= 14.57; *I*^2^= 66%; df = 5; *P* = 0.01). Subgroup analysis was performed according to the type of CHM and showed a significant correlation between the type of CHM and effect size (*P* = 0.002). The forest map showed that the effect of SSA and peoniflorin seemed to be more obvious, although each drug improved the neurological deficit score (*P* < 0.05).

### Subgroup analysis between compound and monomer CHM

**Figure 8:**
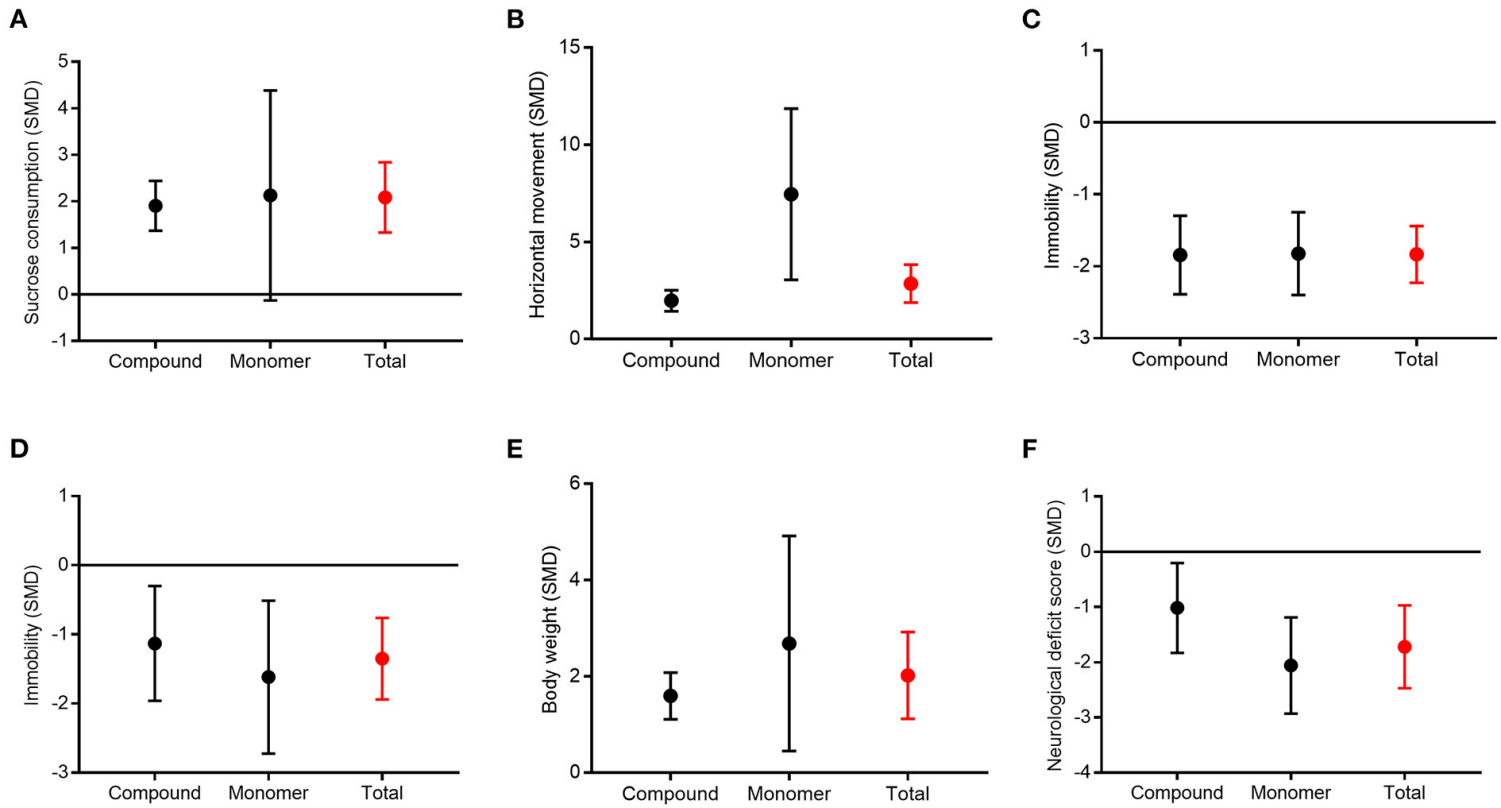
Subgroup analysis between compound and monomer of CHM. Sucrose preference test **(A)**, open-field test **(B)**, forced swimming test **(C)**, tail suspension test **(D)**, body weight **(E)**, and neurological deficit score **(F)**.

Due to the high heterogeneity among the meta-analyses, we further conducted a subgroup analysis according to the form of CHM (compound vs. monomer) (Figure 8). Subgroup analysis showed that compound CHM could significantly improve the sucrose preference test results (*P* < 0.001), with moderate heterogeneity (χ^2^= 35.23; *I*^2^= 55; df = 16; *P* = 0.004). The effect of monomer CHM was similar to that of the control (*P* = 0.06), with considerable heterogeneity (χ^2^= 121.88; *I*^2^= 96; df = 5; *P* < 0.001). However, there were no statistical differences between subgroups (*P* = 0.85; Figure 8A), with no heterogeneity (*I*^2^= 0). For the open-field test, compound and monomer CHM were both associated with increased activity compared to the control group, but monomer CHM was better than compound CHM; this result was statistically significant (*P* = 0.02, Figure 8B), with considerable heterogeneity (*I*^2^= 82.9). For the forced swimming test, tail suspension test, body weight, and neurological deficit score, the effects of both compound and monomer CHM were better than those of the control group. Still, there was no significant correlation between the form of CHM and effect size (*P* = 0.96, *P* = 0.49, *P* = 0.35, *P* = 0.09, respectively; Figures 8C–F). However, the forest plots showed that monomer CHM improved the neurological deficit scores slightly better than compound CHM. Thus, the different forms of CHM may not be the main source of high heterogeneity.

### Publication bias

**Figure 9:**
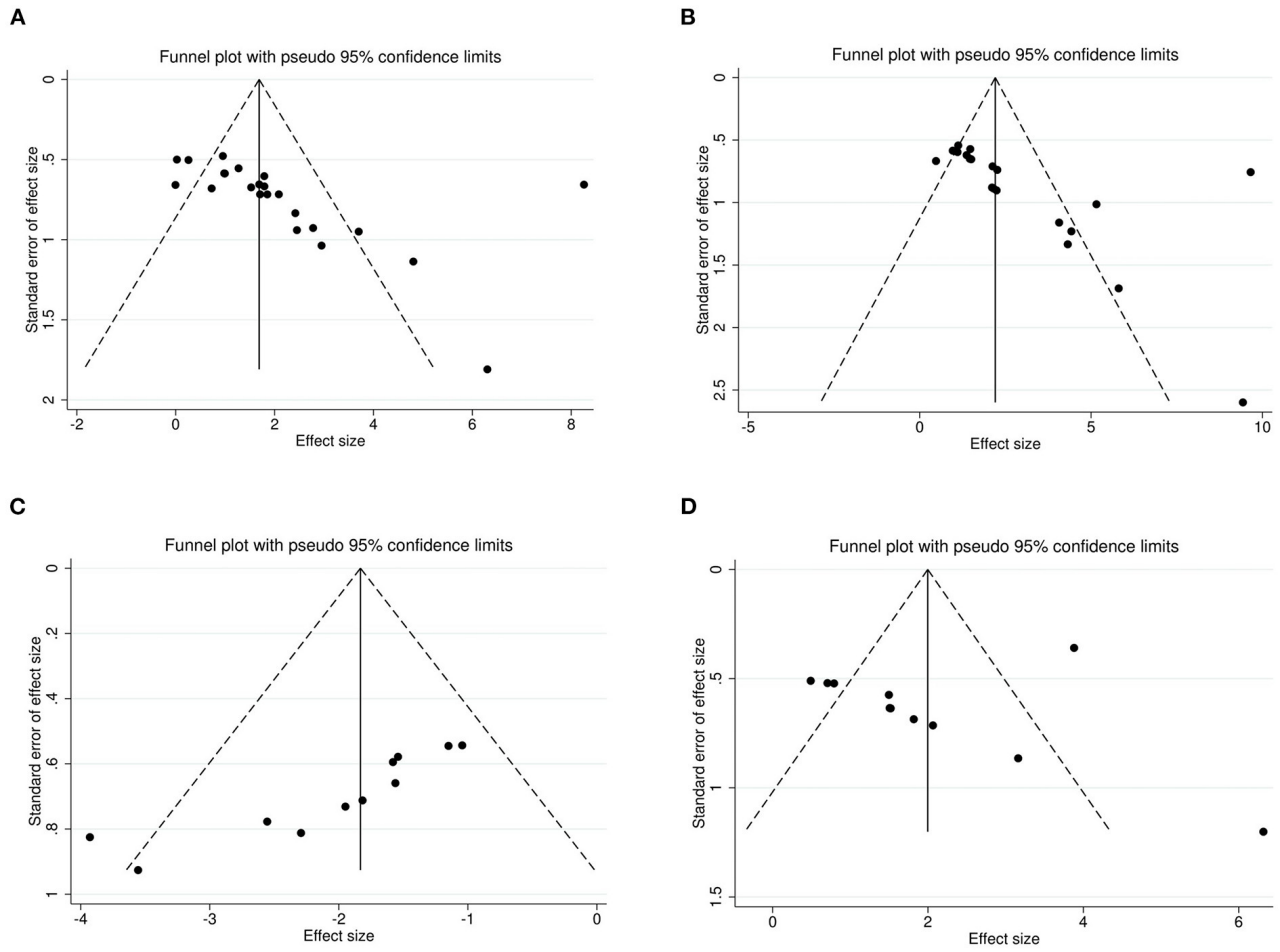
Funnel plots for the sucrose preference test **(A)**, open-field test **(B)**, forced swimming test **(C)**, and body weight **(D)** showing the publication bias.

**Figure 10:**
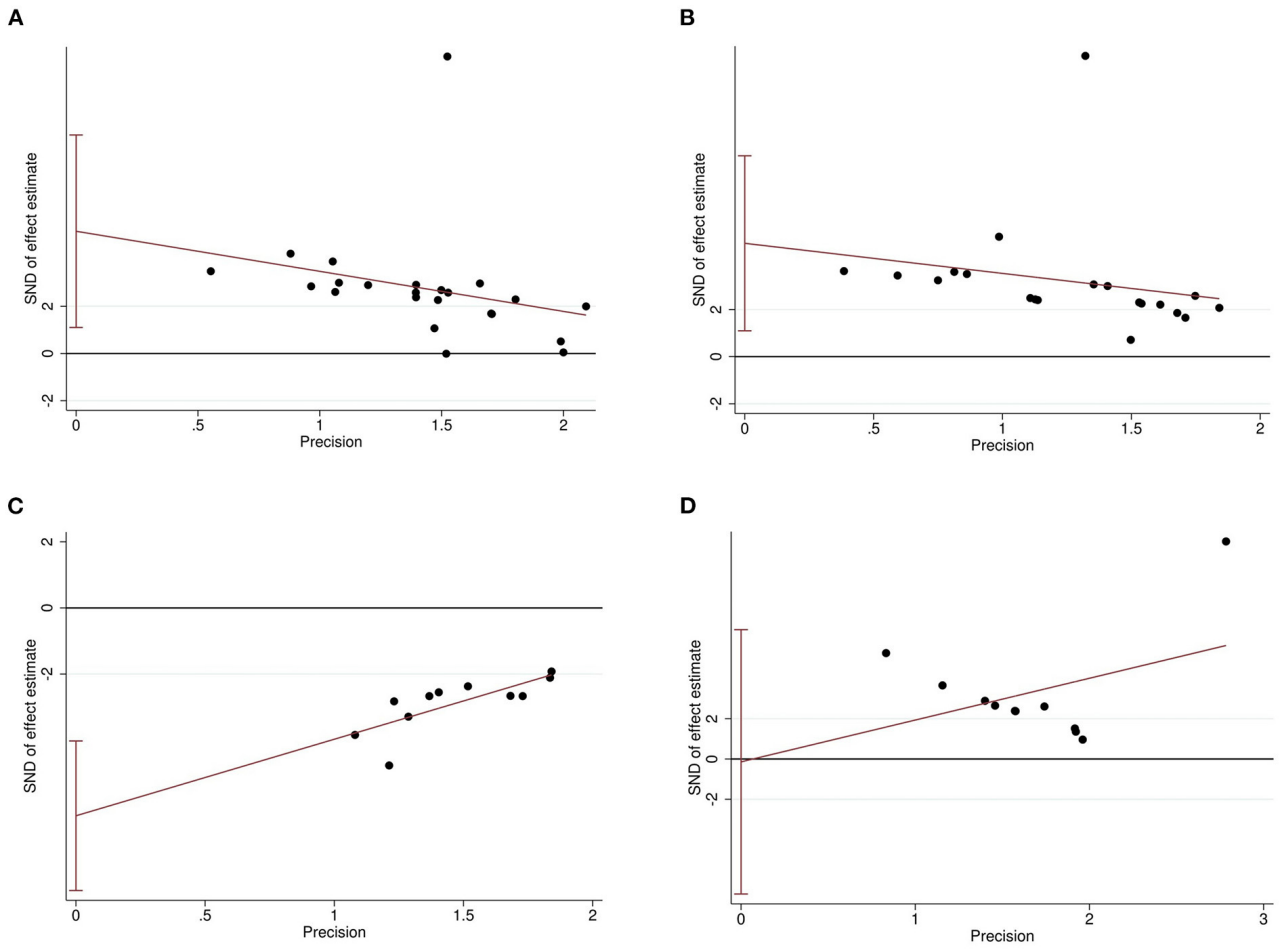
Egger's regression for sucrose preference test **(A)**, open-field test **(B)**, forced swimming test **(C)**, and body weight **(D)** confirming potential evidence for publication bias. The vertical lines represent the 95% confidence interval.

The Egger's test and funnel plots were performed to assess the potential publication bias. Funnel plots of all the outcomes are shown in Figure 9. The Egger's regression test indicated significant publication bias in relation to the sucrose preference, open-field, and forced swimming tests (*P* = 0.015, *P* = 0.014, and *P* < 0.001, respectively; Figures 10A–C), whereas no obvious risk of publication bias was found for body weight (*P* = 0.962; Figure 10D). Few studies assessed the performed neurological deficit scores and performed the tail suspension test; therefore, we did not conduct a publication bias analysis for them.

### Sensitivity analysis

A sensitivity analysis was performed to assess whether any individual study significantly affected the overall meta-analysis results by removing each study one by one (Supplementary Figure S1). All effect sizes fell within the 95% CI of the overall meta-analysis, indicating that no individual study had an excessive influence on the overall results of the meta-analysis. These results show that our statistical results are relatively stable and reliable.

## Discussion

Poststroke depression, the most common psychiatric disorder following a stroke, seriously affects stroke survivors' quality of life and poststroke recovery and also increases their mortality risk (8). The pathogenesis of PSD is not fully understood and is presumably multifactorial. It is thought to involve a combination of various ischemia-induced neurobiological dysfunctions in the context of psychosocial distress (3). The complexity of PSD mechanisms makes prevention and treatment a difficult task. Some western drugs, especially SSRIs, have been proven to be effective. Evidence supports their multi-mechanisms of function. SSRIs are anti-inflammatory agents and enhance neurogenesis through the upregulation of neurotrophins, possibly supported by the stimulation of mitochondrial energy metabolism (3). However, their long-term use is limited due to adverse effects such as bleeding and intracerebral hemorrhage (15). Therefore, it is necessary to explore more effective drugs. Animal studies have found that many CHMs can improve neurological function and depression-like behaviors by regulating neurotrophic factors, hormones, and corresponding signaling pathways and promoting hippocampal neuron regeneration. In this study, we analyzed the effects of CHM on neurological function and depression-like behavior in PSD animals. The pooled results showed that compared with the control group, CHM significantly increased sucrose consumption in the sucrose preference test, increased movement in the open-field test, increased body weight, and significantly reduced immobility time in the forced swimming and tail suspension tests. These results suggest that CHM can significantly improve PSD behavior. In addition, CHM significantly reduced the neurological deficit score, indicating that the neurological function was improved by CHM.

The subgroup analysis according to the type of CHM showed that neurological deficit score, body weight, and sucrose preference test were statistically different among subgroups. Further analysis showed that each drug was effective in terms of the neurological deficit score. The difference among subgroups may be due to the more obvious effect of peoniflorin and SSA. For body weight, the difference among subgroups may have been due to the more obvious effect of peoniflorin, SSA, and the ineffectiveness of MOOs. For the sucrose preference test, the difference among subgroups may be due to the more obvious effect of peoniflorin and the ineffectiveness of MOOs and SSA. The form of CHM was also analyzed as a subgroup, which showed that monomer CHM was better than compound CHM in terms of the open-field test and neurological deficit score. The former was statistically significant, which could be related to the prominent effect of peoniflorin and SSA. We also found that monomers were not effective in terms of the sucrose preference test due to the negative results of MOOs.

The subgroup analysis showed that monomers were more effective in improving neurological function and some depressive symptoms. Peoniflorin was more effective in improving all six indicators, especially the neurological deficit score, body weight, and sucrose preference test results, which were consistent with the results of the previous systematic analysis (37). In the analysis of the improvement effect of SSA on the sucrose preference test, the *P*-value was considered the critical value. Considering the measurement data error and the significant improvement of SSA on the other five indicators, especially the neurological deficit score and body weight, SSA can still be considered a potential drug for treating PSD. This study found that MOOs had no significant improvement in body weight and the sucrose preference test results with no significant heterogeneity. Still, MOOs significantly improved the other five indicators, implying that they may improve depressive symptoms other than anhedonia. We also found that HupA improved the neurological deficit score and sucrose consumption and decreased the immobility time in the forced swimming test. However, due to the small sample size and the small number of studies on monomers, this result should be considered with caution. More studies are needed to confirm this finding. For compound preparations, both the overall and subgroup analysis by drug type or form showed that the results of compound CHM were relatively stable and effective. This could be because there are more components in compounds and, thus, more corresponding targets. Our analysis presents new ideas for the clinical treatment of PSD.

There was heterogeneity in the results of some indicators. Unfortunately, due to the relatively small sample size and the lack of information provided in some studies, we did not conduct more subgroup analyses and did not identify the source of heterogeneity. Indeed, Egger's test results and funnel plots suggest that publication bias exists in the current study. The sensitivity analysis suggested that the results were stable and reliable, but our findings should be interpreted with caution due to the small number of studies included.

This study has several limitations. First, we only included studies published in English, which may have led to publication bias. Second, only a limited number of studies were included, and the total sample size was small. Third, additional subgroup analyses could not be performed because of limited data. Fourth, we failed to get all the original data, and some data were measured using ImageJ. Therefore, some errors may have been introduced that could affect the accuracy of the results. Finally, the current studies only investigated the efficacy of CHM on PSD, and the safety of CHM has not been analyzed. In the future, more studies are needed to confirm the safety and efficacy of CHM in the treatment of PSD.

## Conclusion

Our results showed that CHM could significantly improve the depression-like behaviors and neurological function of animals with PSD. CHM could be potentially used in the treatment of PSD. However, this study is a meta-analysis based on animal studies. More high-quality preclinical trials and clinical studies are needed to verify our results before CHM can be used in clinical settings.

## Data availability statement

The original contributions presented in the study are included in the article/Supplementary material, further inquiries can be directed to the corresponding author.

## Author contributions

LL and BH: conception and design, analysis and interpretation of data, drafting the manuscript, and statistical analysis. YuW, TL, and GS: searching the literature. LL and YG: screening of titles and abstracts and full-text data extraction. YaW, YZ, and TZ: critically revising the manuscript. All authors have read and agreed to the published version of the manuscript.
