# Supplementary material for: Efficacy of Chinese herbal medicine on poststroke depression in animal models: A systematic review and meta-analysis
Source: Front Neurol. 2023 Jan 9;13:1095444. doi: 10.3389/fneur.2022.1095444 (PMC9870325; doi:10.3389/fneur.2022.1095444)
Supplement: Supplementary file 1 [file Data_Sheet_1.PDF]

SUPPLEMENTARY MATERIALS

Search Strategy

1. Search strategy used in *PubMed* database:

#1. animal OR animals OR rat OR rats OR mouse OR mice[Title/Abstract] Items found: 3,487,902

#2. stroke[Title/Abstract] Items found: 291,778

#3. depression[Title/Abstract] Items found: 402,584

#4. #2 AND #3 Items found: 8,801

#5. Chinese medicine OR Chinese herb OR herbal OR natural drug OR Natural Product OR traditional Chinese OR formula[Title/Abstract] Items found: 195,234

#6. #1 AND #4 AND #5 Items found: 20

Final Result: 20 (By Sep 24, 2022)

2. Search strategy used in *Web of Science* database:

#1. AB=(stroke)   Items found:264,631

#2. AB=(depression)   Items found:370,247

#3. #1 AND #2   Items found:7,560

#4. AB=(rat OR rats OR mouse OR mice OR animal OR animals)   Items found:2,763,542

#5. AB=(Chinese medicine OR Chinese herb OR herbal OR natural drug OR natural product OR traditional Chinese OR formula)   Items found:652,399

#6. #3 AND #4 AND #5   Items found:20

Final Result: 20 (By Sep 24, 2022)

3. Search strategy used in *Cochrane Library* database:

#1. ABS- TITLE-KEY=stroke Items found:78,370

#2. ABS- TITLE-KEY=depression Items found:91,549

#3. #1 AND #2 Items found:3,607

#4. ABS-TITLE-KEY=(rat):ab,ti,kw OR (rats):ab,ti,kw OR (mouse):ab,ti,kw OR (mice):ab,ti,kw OR (animal):ab,ti,kw OR (animals):ab,ti,kw Items found:37,979

#5. ABS-TITLE-KEY=(Chinese medicine):ab,ti,kw OR (Chinese herb):ab,ti,kw OR (herbal):ab,ti,kw OR (natural drug):ab,ti,kw OR (Natural Product):ab,ti,kw OR (traditional Chinese):ab,ti,kw OR (formula):ab,ti,kw Items found:43,925

#6. #3 AND #4 AND #5 Items found:20

Final Result: 20(By Sep 25, 2022)

4. Search strategy used in *Embase* database:

#1. ABS-TITLE=('stroke') Items found:543,030

#2. ABS-TITLE =('depression') Items found:809,572

#3. #1 AND #2 Items found:19,648

#4. ABS-TITLE=('rat':ab,ti OR 'rats':ab,ti OR 'mouse':ab,ti OR 'mice':ab,ti OR 'animal':ab,ti OR 'animals':ab,ti) Items found:4,285,385

#5. ABS-TITLE=('Chinese medicine':ab,ti OR 'Chinese herb':ab,ti OR 'herbal':ab,ti OR 'natural drug':ab,ti OR 'natural product':ab,ti OR 'traditional chinese':ab,ti OR 'formula':ab,ti) Items found:282,378

#6. #3 AND #4 AND #5 Items found:41

Final Result: 41(By Sep 25, 2022)

Hand search in *PubMed* database:N=3 (By Sep 25, 2022)

Final Result: 104

Supplementary Figure 1

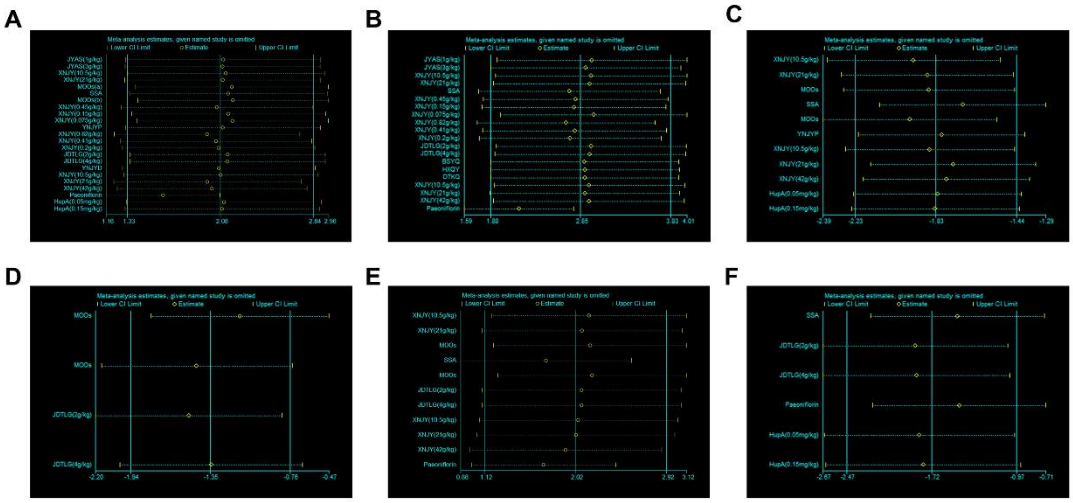

Figure S1. Sensitivity analysis for sucrose preference test (A), open-field test (B), forced swimming test (C), tail suspension test (D), body weight (E) and neurological deficit score (F) evaluating the robustness of the results. The horizontal yellow bars represent the potentially minimum and maximum 95% confidence interval and the horizontal green bars represent the actual 95% confidence interval of the pooled estimate of efficacy.
